# Institutional Notes and News

**Published:** 1910-09-17

**Authors:** 


### Institutional Notes and News.

**Cumberland Infirmary.**

The chief matter of interest at the recent quarterly meeting of the Governors of the Cumberland Infirmary was the presentation and adoption of the augmentation of Income Committee's report. That report, a model of brevity, was explained by the Chairman, Major Ferguson, to mean that £1,000 a year more is needed if the Infirmary is to meet fully and adequately the demands of the growing neighbourhood. The need for further support is apparent from the Treasurer's report for the recent quarter in which we find that £1,549 was spent, while only £954 was received. It is true that this discrepancy will be reduced somewhat when the money from the Saturday and Sunday Funds has been received, but against this must be set the balance on the wrong side of £715 with which the year started, a balance, we learn, unfortunately likely to reach four figures at the end of this year. It was decided, therefore, to apply a legacy of £200 received last May to the current account. When it is remembered that Cumberland alone provided enough patients to keep the beds full, while South Scotland does not hesitate to make a claim upon them, it is clear that the need for a wider circle of subscribers is genuine and must be met.

**The Dental Hospital at Sydney.**

The Queen Victoria Memorial Hospital, entirely officered and managed by women, finished the year with a credit balance of £348. The Sydney dentists are building a new dental hospital, finding the old one cramped and unsuitable, though 50,000 cases have been treated in it. At the foundation-stone laying ceremony, Professor Anderson Stuart said that "the University viewed the dental hospital with very great satisfaction. It would be an important aid to dental education, and in a few months he hoped they would have a dental faculty."

The State Treasurer again has been exercising his authority, and is withholding from one of the principal country hospitals, the Gippsland, at Sale, a balance of the State grant on the ground that certain renovation work has not been effected. The Committee have no available funds for this work and are in urgent need of the withheld money to meet outstanding accounts; also, they question the State Treasurer's right so to act with regard to money voted by Parliament for a special purpose, and it appears that a pretty quarrel is brewing.

**Australian Hospital Reports.**

All public institutions have been busy issuing their annual reports on the end of the financial year (June 30). The Melbourne Hospital finds itself better off than was anticipated, owing to special sums received. "In addition, £600 has been received from bequests which, under former conditions, would have gone to the endowment account (instead of maintenance account)." This alteration in the rules was made in accord with a suggestion of the State Treasurer (Mr. Watt). The cost of working the hospital had been materially decreased, but the economies effected had been swallowed up by the enhanced price of coal and the cost of furnishing rooms for the house matron and female cooks. It is expected that a commencement will be made with the new hospital buildings by the middle of October. The Government grant is to be increased by £1,000.

The Alfred Hospital still finds itself badly off. In response to a question, the secretary furnishes the following explanation: "The falling-off I referred to was from the whole body of in-patients. The privileges formerly accorded those who paid 30s. a week were treatment in a ward containing not more than four beds and visits from their friends *any* day between 3 and 5 P.M., instead of limitation to two days. These privileges meant that, whereas we now only get 15s. or 20s. a week, we then got 30s., as people made an effort to secure these privileges. For years ending June 30, 1905-6-7, our private contributions exceeded £1,900; in 1908, £1,871; 1909, £1,682. For the year just ended the sum has risen again to £1,916; but the total number of in- and out-patients treated has also gradually risen, from 9,564 in 1904-5 to over 12,000 in 1909-10."

The report of the Homœopathic Hospital was characterised by Sir Arthur Snowden, who presided at the annual meeting, as "one of the most satisfactory he had ever seen." A great deal of building has been done this year, and yet the institution is not in debt. In spite of the State Treasurer's refusal to donate money towards a children's ward, on the ground that there is a children's hospital, the authorities went on with it, and were enabled, by the gift of £5,000 from an anonymous donor, not only to build but to furnish it. New quarters for nurses have also been erected and furnished.

**Halifax Eye, Ear and Throat Hospital.**

The Halifax Eye, Ear, and Throat Hospital has just held its annual meeting, and the submission of the balance sheet shows that it begins its new year again with a balance in hand. The number of in-patients treated was 74, and 2,202 patients were treated in the out-patients' department. The latter had a total attendance of 27,170. It was only to be expected therefore that, as Mr. Alderman J. Collinson pointed out, they had been compelled to buy an unusually large quantity of spectacles and new instruments. Mr. Collinson said also that the balance in hand was a proof that the patients were grateful for the treatment, and had subscribed more liberally than ever to the upkeep of the institution. The finance committee, moreover, offered its grateful thanks to the Halifax Corporation for once more exempting the hospital from the payment of borough rates. A new matron, Mrs. Coates, from the Manchester Royal Infirmary, has been appointed in succession to Mrs. Crossley, who had worked faithfully for the hospital for twelve years. Further accommodation for the in-patients is wanted, and it is hoped that subscribers will come forward liberally enough to enable another house to be taken.

**The London Hospital.**

At the quarterly meeting of the board of governors of the London Hospital, held last week, the Chairman announced that the grant from the Hospital Sunday Fund amounted to £5,941. Lord Tredegar, the hospital's landlord, has presented the hospital with a freehold of Tredegar House for the training of nurses. Mr. Raphael has given £1,000 to name a ward. Mr. Hora, whose generosity is constant, has deposited securities sufficient to raise his endowment to £520 a year, a sum which will relieve the hospital from the charges borne since the opening of the Marie Celeste wards. Dr. Murphy, a former student, had acquired certain property in South Africa which he left in equal shares to Guy's and to the London. This, known as the Murphy Estate, has to be sold, without reserve, this month by the executors, according to the law in South Africa. Considerable difficulty has been experienced in ascertaining its position, value, and chance of a purchaser.

**Hull Royal Infirmary.**

His Majesty's Inspectors of Factories for some time past have been casting rather jealous eyes on hospitals, and we are sorry therefore to record that the Hull Royal Infirmary has been fined £40, including costs, for an offence under the Factory Act. It appears that a woman named Kate Edwards has had her right hand injured seriously owing to the fact that the intake of one of the laundry calendars had not been fenced. Mr. J. Owner, his Majesty's Inspector, at whose instance the summons was taken out, submitted that the hospital had been warned repeatedly. The offence was admitted, but the authorities of the infirmary urged that for many years there had been no accident. Only a few months ago a case was reported in these columns in which it was decided that a hospital laundry was covered by the Factory Acts. That being so, we hope that other hospitals will take warning from the Hull Royal Infirmary and comply readily with the regulations. If it is true that the infirmary authorities were warned several times before the summons was taken against them, they cannot expect much sympathy. Such cases are particularly regrettable, because hospitals ought to be the very last places where the chance of accident to the institutional staff is not guarded against to the full. As regards the injured woman, the Magistrate directed that she should receive such benefit as the Home Secretary might determine. We have no doubt that the hospital has done for her already so much as to make any action of the Home Secretary's a work of supererogation.

**East Grinstead Cottage Hospital.**

The East Grinstead Cottage Hospital has now the satisfaction of seeing its extension completed by the formal opening of the two enlarged wards on September 1. This means that four extra beds have been added by the generosity of Mrs. Hyde's offer of £500 in memory of her late husband. A private ward has been added also, the neighbourhood having come forward in support. Mr. Mathews has acted as honorary architect, and received the thanks of the chairman for his excellent work, and to Mrs. Hookham and Miss Hyde a resolution of thanks was passed for their help respectively towards the furnishing and maintenance of the hospital. An appeal was addressed to the tradesmen of East Grinstead to bear their share in the increased expenses of upkeep which the extension necessarily causes, for East Grinstead, which was the fourth town in England to have its cottage hospital, must not fall behind now, when there is need for a renewal of effort. After a vote of thanks had been carried thanking Mr. White for his generosity in clearing the general account, the new wards were opened and inspected by the visitors.

**Mr. Herbert Samuel at Benenden.**

To supplement the account we gave last week of the aims of the Benenden Sanatorium, we give this week details of the opening of the new wing. On Monday last the Right Hon. Herbert Samuel, the Postmaster-General, accompanied by the Right Hon. Sydney Buxton, officially opened the new twenty-bed pavilion attached to the National Sanatorium for Consumptives at Benenden, Kent. This pavilion has been built and entirely equipped by means of subscriptions of sums from 1d. to 5s., voluntarily given by Post Office men and women, for whose sole benefit the building has been erected. At the luncheon given in the recreation-room previous to the opening, the Postmaster-General said that the Post Office Sanatorium Society now numbered about 45,000 members. He greatly admired the work of the Sanatorium, which was carried on on the great principle of self-help. He regretted, however, that the new building would be opened with a debt of £500, but he hoped that this amount would be very quickly cleared by increased membership and subscriptions. He complimented Mr. C. H. Garland, the founder and chairman of the Sanatorium, upon the excellent work and progress made at Benenden. Mr. Buxton, Mr. Waldorf Astor, and Mr. West (the Treasurer of the Association), also spoke, and Mr. Garland replied, giving a full detailed account of the history and work of the organisation. He then handed Mr. Samuel a case containing a replica in silver of the key of the new building. After the opening ceremony the whole of the sanatorium was thrown open for public inspection.

